# Gonorrhea Management in High‐, Limited‐ and No‐Resource Settings: Implications in the Context of Antimicrobial Resistance

**DOI:** 10.1111/ijd.70040

**Published:** 2025-08-27

**Authors:** Andrei Tanasov, Maciej Pastuszczak, George‐Sorin Tiplica

**Affiliations:** ^1^ Carol Davila University of Medicine and Pharmacy Bucharest Romania; ^2^ 2nd Department of Dermatology Colentina Clinical Hospital Bucharest Romania; ^3^ Department of Dermatology Medical University of Silesia Katowice Poland

**Keywords:** antimicrobial resistance, doxycycline post‐exposure prophylaxis, gonorrhea, high‐resource settings, low‐ and middle‐income countries, migrant health, *Neisseria gonorrhoeae*, point‐of‐care testing, sexually transmitted infections, syndromic management

## Abstract

Gonorrhea, a common sexually transmitted infection caused by *Neisseria gonorrhoeae* , represents an escalating global public health threat due to antimicrobial resistance. The current review explores diverse approaches around the globe to gonorrhea management across various settings, with a focus on diagnostic strategies, treatment practices, resistance surveillance, and new trends, such as point‐of‐care tests or doxycycline post‐exposure prophylaxis. High‐resource settings benefit from proper infrastructure, advanced diagnostics, screening programs, surveillance, access to effective treatment, and test‐of‐cure protocols. Nonetheless, rising incidence and resistance rates persist. In contrast, limited‐resource settings often depend on syndromic management, which lacks sensitivity and fails to identify asymptomatic infections. The low diagnostic capacity and poor access to resistance‐guided therapy contribute to inappropriate antimicrobial use. Migrants, incarcerated individuals, and those in vulnerable contexts face additional barriers. These disparities undermine the global control efforts and enable the emergence of drug‐resistant strains. Addressing these gaps requires a multifaceted, equity‐focused approach: strengthening diagnostic capacity, expanding access to molecular testing and surveillance, tailored interventions for the local contexts, and promoting antibiotic stewardship in both policy and practice.

## 1 Introduction

Gonorrhea is a common sexually transmitted infection (STI) caused by *Neisseria gonorrhoeae* (NG), which primarily manifests as urethritis, cervicitis, pharyngitis, proctitis, conjunctivitis, and can occasionally result in systemic dissemination. This infection poses a significant risk of serious complications, including pelvic inflammatory disease and infertility. According to the World Health Organization (WHO) Global Progress Report on Human Immunodeficiency Virus (HIV), Viral Hepatitis and STIs, 82.3 million new cases of NG infections among people 15 to 49 years old occurred globally in 2020 [1]. Due to the alarming rise in antimicrobial resistance, NG has been designated by the WHO as one of the high‐priority pathogens of global public health concern [2].

The presence of asymptomatic NG infections, particularly at extragenital sites: pharynx and rectum, contributes to sustained transmission and poses a significant challenge for diagnosis, particularly among under‐recognized or underserved high‐risk populations. Concurrently, these undetected reservoirs may facilitate the emergence and spread of antimicrobial‐resistant strains, further complicating control and treatment efforts [3].

While doxycycline post‐exposure prophylaxis (DoxyPEP), typically administered at 200 mg within 24 to 72 h after sexual contact, shows promise in preventing certain non‐gonococcal bacterial STIs [4], its widespread use raises significant concerns regarding its potential to accelerate antimicrobial resistance in NG [5, 6].

These trends manifest unevenly across the globe, reflecting disparities in healthcare infrastructure, diagnostic capacity, and access to treatment. Guidelines from the Centre for Disease Control and Prevention (CDC), WHO, and the International Union against Sexually Transmitted Infections‐Europe (IUSTI‐Europe) assume the availability of local antimicrobial resistance data, which is often lacking. In many regions, susceptibility testing is unavailable, access to recommended antibiotics may be limited, and test‐of‐cure protocols are inconsistently implemented. These gaps highlight the need for context‐specific strategies.

This review examines the global landscape of gonorrhea management, with a focus on how variations in diagnostic capacity, treatment access, and public health systems affect infection outcomes, the development of antimicrobial resistance, and the effectiveness of control strategies. The review explores how the differences in healthcare infrastructure, public health priorities, and socioeconomic landscapes between high‐ and low‐income countries influence gonorrhea management strategies and the trajectory of antimicrobial resistance.

## 2 Methods

We conducted a narrative review by searching PubMed, Web of Science, Google Scholar, using combinations of keywords such as: “ *Neisseria gonorrhoeae* ” “gonorrhea” “antimicrobial resistance” “surveillance” “point‐of‐care testing” “DoxyPEP” “extragenital screening” “test‐of‐cure” “low‐resource settings” “syndromic management” “migrants” “partner notification” “crisis settings”. We included studies published in English from 2020 to 2025, as well as publicly available reports from global health agencies and regulatory bodies, including the WHO, CDC, European Centre for Disease Prevention and Control (ECDC), and IUSTI.

## 3 Gonorrhea Surveillance

Effective surveillance is essential for targeted gonorrhea control, yet it remains uneven across resource settings: well‐established in high‐income countries, underdeveloped or absent in many low‐ and middle‐income regions.

Recent European surveillance data from 2023 indicate a continued upward trend in the European Union/European Economic Area (EU/EEA) region: a total of 96,969 confirmed cases were reported, corresponding to a crude notification rate of 25 cases per 100,000 population, which represents the highest rate since the initiation of surveillance, and a 31% increase compared to the previous year. A substantial proportion of infections occurred among men who have sex with men (MSM), accounting for 58% of cases with a documented transmission route. The 25 to 34‐year‐old age group represented the largest share of infections (37%). Notably, young females also experienced a marked rise in incidence: among women aged 20 to 24 years, the number of cases increased by 46% relative to 2022, and among those aged 25 to 34 years, by 45% [7].

Extensively drug‐resistant NG strains have recently been identified in the European space [8], characterized by high‐level resistance to azithromycin (MIC > 256 mg/L) alongside resistance to ceftriaxone, cefixime, cefotaxime, ciprofloxacin, and tetracycline [9]. Cases have been reported of individuals harboring distinct NG strains at different anatomical sites, with varying resistance profiles [10].

Addressing gonococcal AMR relies on coordinated global surveillance efforts (Table 1). The WHO Gonococcal Antimicrobial Surveillance Program (GASP) operates through reference laboratories linked with multiple regional and national initiatives, including Euro‐GASP (Europe), GISP (United States), GRASP (United Kingdom), AGSP (Australia), and aims to collect at least 100 representative NG isolates per country per year, using quantitative methods to determine the minimum inhibitory concentrations (MICs), predominantly using the established reference values of the European Committee on Antimicrobial Susceptibility Testing (EUCAST) [11].

**TABLE 1:** Prevalence of antimicrobial resistance in *Neisseria gonorrhoeae* isolates across selected countries.

Country	Isolates number	Percentage of strains resistant to antibiotics				Programe and year
		Ceftriaxone	Cefixime	Azithromycin	Ciprofloxacin	
Western Pacific						
Australia	8199	0.51	N/A	3.9	63.3	AGSP, 2022
Philippines	700	0	0	0	94	EGASP, 2023
Viet Nam	260	20	30	7	N/A	
Africa						
Malawi	122	0	0	0	77	
South Africa	341	0	0	3	N/A	
Uganda	298	0	0	0	100	
Zimbabwe	18	0	0	0	76	
South‐East Asia						
Cambodia	274	15	53	21	99	
Indonesia	105	0	0	0	97	
Thailand	373	0	0	1	98	
Europe						
Austria	377	0.26	0.8	27.9	76.9	EURO‐GASP, 2022
Belgium	200	0	0.5	43	56.5	
Bulgaria	12	0	0	16.7	33.3	
Czech Republic	111	0	0	21.6	59.5	
Denmark	135	0	0	2.2	32.6	
Estonia	8	0	0	16.7	75	
Finland	91	0	0	13.2	52.7	
France	220	0	0	12.3	67.7	
Germany	200	0.5	0.5	24.5	64	
Greece	100	0	0	25	66	
Hungary	122	0	1.6	14.8	77	
Iceland	63	0	1.6	34.9	57.1	
Ireland	294	0	0.3	46.9	59.5	
Italy	100	0	1	22	84	
Malta	61	0	0	13.1	85.2	
Netherlands	572	0	0	35.1	63.6	
Norway	827	0	0.2	21	59.9	
Poland	15	0	0	40	66.7	
Portugal	110	0	0	68.2	68.2	
Slovakia	80	0	0	13.3	72.5	
Slovenia	285	0	0	11.2	87.4	
Spain	213	0	0	21.1	75.6	
Sweden	200	0	2	43	66	
United Kingdom	1460	0	0.8	6.8	58.6	GRASP, 2022
Americas						
USA	3684	0.02	0.13	4.07	36.83	GISP, 2022

Abbreviations: AGSP, Australian Gonococcal Surveillance Programme; EGASP, Enhanced Gonococcal Antimicrobial Surveillance Programme; EURO‐GASP, European Gonococcal Antimicrobial Surveillance Programme; GISP, Gonococcal Isolate Surveillance Project; GRASP, Gonococcal Resistance to Antimicrobials Surveillance Programme; N/A, not available.

Beyond Europe, the epidemiological landscape and resistance patterns show significant heterogeneity. The WHO's Enhanced Gonococcal Antimicrobial Surveillance Programme (EGASP) provides critical AMR insights into regions with historically limited data, along with demographics, behavioral data, sexual history, and antibiotic use [12]. The 2023 EGASP report included a total of 3498 episodes of urethritis reported across nine countries from the WHO Western Pacific Region (Philippines, Cambodia, Viet Nam), the WHO African Region (South Africa, Uganda, Malawi, Zimbabwe), and the WHO South‐East Asia Region (Thailand and Indonesia), of which 2502 NG isolates were confirmed by culture. Antimicrobial susceptibility testing was performed for the vast majority, revealing that 4% of the overall isolates showed resistance to ceftriaxone and 96% to ciprofloxacin [13]. There are certain hotspots; for instance, Viet Nam reported a ceftriaxone resistance rate of 26.9% in 2023 [14]. This global variation in AMR profiles, detailed in Table 1, underscores the necessity of tailoring treatment guidelines to local resistance data.

On the African continent, gonorrhea antimicrobial resistance surveillance remains limited, with only a small number of countries reporting data to the WHO (such as only South Africa, Malawi, Ghana, and Madagascar in 2016). A systematic review of available reports from sub‐Saharan Africa reveals a consistently high prevalence of resistance to commonly used antibiotics, and, alarmingly, strains resistant to ceftriaxone have been identified across all subregions except West Africa [15]. The Eastern Mediterranean Region also faces significant gaps in gonorrhea antimicrobial resistance surveillance, one available dataset from Qatar (2017–2020) reporting resistance rates of 64.7% for ciprofloxacin, 50.7% for tetracycline, 30.8% for benzylpenicillin, and 4.1% for azithromycin, while in Morocco, surveillance conducted in 2013 identified high levels of ciprofloxacin resistance among men with gonorrhea (86.8%), prompting a shift in national treatment guidelines from ciprofloxacin to ceftriaxone 250 mg [11].

While high‐income countries may contribute regularly to surveillance programs, many low‐ and middle‐income countries lack the infrastructure to participate. This imbalance limits the global understanding of resistance patterns and creates blind spots in public health response.

## 4 Gonorrhea in High‐Resource Settings

**FIGURE 1:**
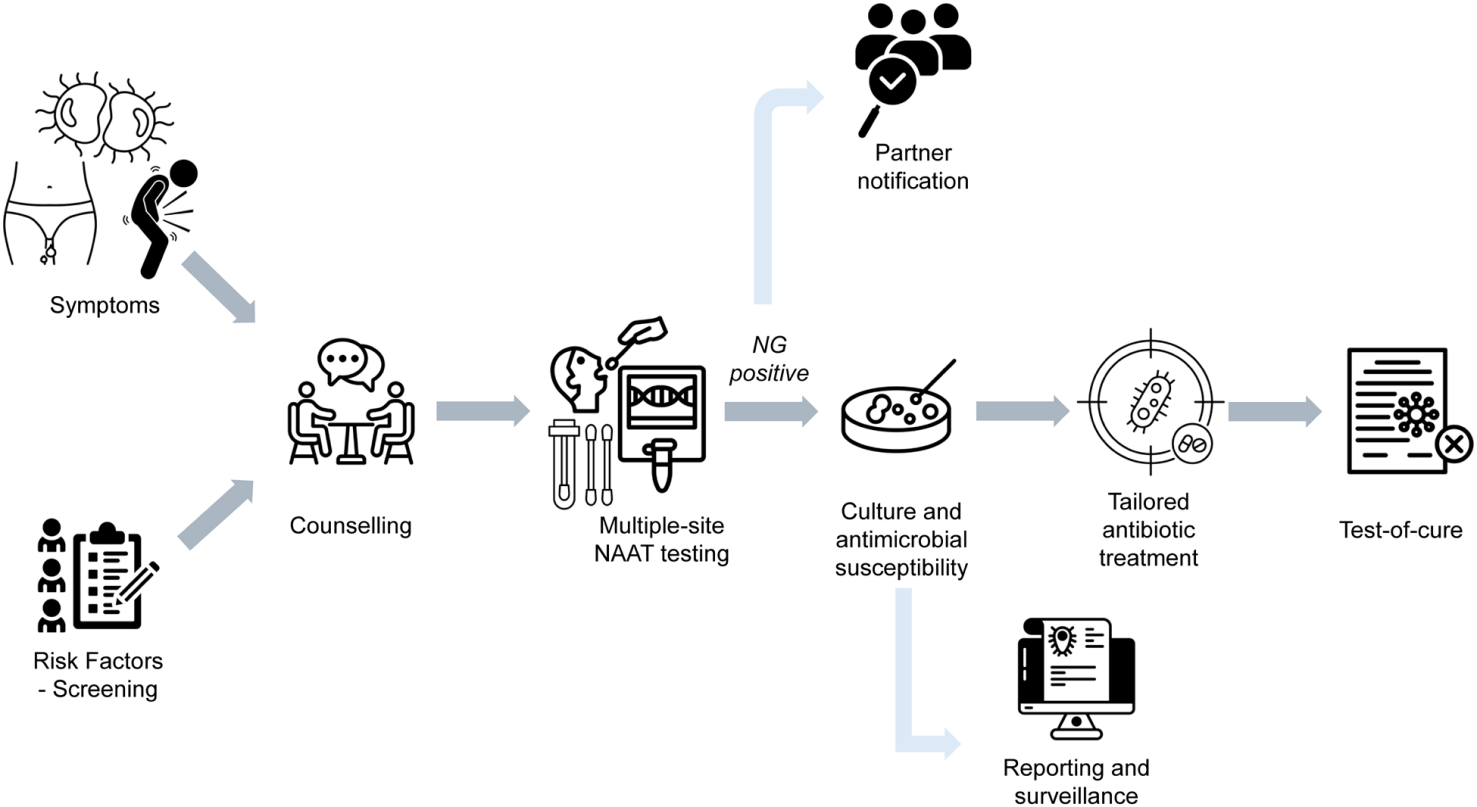
Gold standard of gonorrhea management. Arrows indicate the clinical workflow. The comprehensive management approach incorporates screening strategies, multisite testing, antimicrobial susceptibility surveillance, tailored treatment with test‐of‐cure, as well as partner notification. Concurrent sampling for NAAT and culture is advisable. NAAT, nucleic acid amplification test.

In high‐resource settings, having specialized STI clinics and major hospitals with facilities to develop robust antimicrobial susceptibility surveillance, gonorrhea management benefits from comprehensive diagnostic and treatment capabilities. This allows full‐spectrum testing, including antimicrobial susceptibility, and facilitates advanced approaches such as targeted screening or test‐of‐cure protocols (Figure 1).

### 4.1 Screening and Diagnosis

Beyond clinical symptomatology, routine gonorrhea testing is essential for individuals in specific risk categories, including patients diagnosed with other STIs, individuals under 25 years of age, MSM, and those with multiple or casual sexual partners [16]. Considering rising gonorrhea rates among MSM, particularly extragenital infections, the burden in this population has become increasingly well‐documented, and as a result, multiple national guidelines in high‐resource settings recommend routine triple‐site screening—urethral/urine, pharyngeal, and rectal every 3 to 6 months [17]. In a study involving 3938 MSM undergoing triple‐site screening, 11.4% (95% confidence interval [CI]: 10.3%–12.2%) were found positive for gonorrhea at one or more anatomical sites, with approximately 80% of these infections being asymptomatic [18]. In a cohort of people living with HIV, only 0.25% (1/605) of urogenital samples tested positive for NG, whereas positivity was higher in oropharyngeal (4.5%; 13/291) and rectal (5.8%; 16/278) specimens [19].

The median duration of asymptomatic NG carriage has been estimated at 16.3 weeks (95% CI: 5.1–19.7 weeks) in the pharynx and 9 weeks (95% CI: 3–12 weeks) in the rectum, based on data from a cohort of 140 MSM [20, 21]. Modeling studies suggest that extragenital screening of all MSM presenting for urogenital testing could reduce site‐specific gonorrhea prevalence by an average of 42%–50% [22], however, it has as well been proposed that reducing screening frequency, e.g., from quarterly to annually, may offer benefits such as lower healthcare costs, reduced antimicrobial use, and potentially slower development of antimicrobial resistance [17]. Testing from multiple anatomical sites requires additional costs and resources.

While often discussed in the context of expanding access in low‐resource settings, point‐of‐care tests (POCTs) play an increasingly vital role in high‐income countries. Here, their primary value lies in the immediacy of results, providing results for NG and CT in 90 min. This allows for immediate, diagnosis‐guided treatment during a single clinical encounter, particularly impactful in settings with high patient turnover and potential for loss to follow up, such as emergency departments or busy primary care clinics. In an emergency department study, point‐of‐care testing for CT and NG led to significantly more accurate management than standard syndromic care: inappropriate treatment occurred in only 5% of point‐of‐care cases versus 36% with standard care (*p* < 0.001), largely due to reduced overtreatment enabled by rapid results [23].

### 4.2 Treatment and Test‐of‐Cure

The 2020 IUSTI‐Europe guideline for the management of gonorrhea recommends dual antimicrobial therapy with ceftriaxone 1 g and azithromycin 2 g for the treatment of uncomplicated cases, when antimicrobial susceptibility is unknown. The guideline also emphasizes the importance of obtaining culture specimens to guide therapy and performing a test‐of‐cure after the antibiotic treatment [16].

The United States CDC currently recommends ceftriaxone as monotherapy for the treatment of uncomplicated gonococcal infections of the cervix, urethra, or rectum in adults and adolescents, with the recommended dose 500 mg of ceftriaxone in a single dose for individuals weighing < 150 kg, and 1 g for those weighing 150 kg or more. If *Chlamydia trachomatis* (CT) infection has not been excluded, concurrent treatment with doxycycline 100 mg orally twice daily for 7 days is advised; azithromycin has been removed from the standard gonorrhea treatment regimen due to a rapid increase in resistance and concerns about promoting resistance in other pathogens, such as *Mycoplasma genitalium* . The ceftriaxone dosage adjustment has been supported by pharmacokinetic modeling, which demonstrates that a 500 mg dose maintains free ceftriaxone concentrations for 49.9 h, exceeding the estimated 20 to 24 h threshold required for cure, even at pharyngeal sites. This change has been further justified by surveillance data showing that fewer than 0.1% of circulating strains exhibit decreased susceptibility to even 250 mg of ceftriaxone [24, 25].

Several European countries have shifted their national guidelines towards ceftriaxone monotherapy, in the context of increasing azithromycin resistance. For instance, the 2025 guideline from the UK's British Association for Sexual Health and HIV (BASHH) recommends ceftriaxone 1 g intramuscularly as the sole first‐line empirical treatment, a significant departure from previous dual therapy recommendations [26]. Similarly, German urethritis guidelines updated in 2025 prefer ceftriaxone monotherapy [27].

A test of cure should be performed in all patients treated for gonorrhea, between 3 and 7 days posttreatment if symptoms persist, or at 2 weeks for asymptomatic individuals, to confirm clearance of infection. If the Nucleic Acid Amplification Test (NAAT) remains positive, culture is essential to assess for antimicrobial resistance [16]. Studies have shown that return rates for test‐of‐cure visits are variable, and positive results are interpreted as either reinfection or false positives, even for cases involving isolates with reduced antibiotic susceptibility [28] or pharyngeal infections [29]. In other settings, such as among people living with HIV and diagnosed with oropharyngeal gonorrhea, test‐of‐cure rates remain concerningly low, even when supportive interventions such as home testing kits are provided: in one study, only 23 out of 223 patients (10.3%) completed repeat testing within the recommended 7–14‐day window [30]. Additionally, studies have shown that a substantial proportion of patients engage in sexual activity before completing their test‐of‐cure; for example, among 540 study participants, 37% reported sexual contact during this period, with 88% of those individuals reporting inconsistent condom use [31].

The DoxyPEP trial among MSM and transgender women demonstrated a significant reduction in STIs, including syphilis and CT infection, and a more modest or variable reduction in gonorrhea, when doxycycline was taken within 72 h after condomless sex [5]. Its use is growing in high‐income countries. However, resistance concerns remain: an increase in the percentage of tetracycline‐resistant NG was observed among doxycycline users (27% of the NG isolates vs. 24% in the nonusers), underlining the importance of AMR surveillance prior to widespread implementation [5, 32]. Whole‐genome sequencing of 2375 Euro‐GASP isolates identified links between tetracycline resistance mutations and the mosaic *penA‐60* allele, which confers resistance to extended‐spectrum cephalosporins. These findings raise concern that doxycycline use may promote ceftriaxone‐resistant strains [33, 34].

### 4.3 AMR Testing

The gonorrhea IUSTI‐Europe guidelines mention the importance of culturing the gonorrhea isolates to assess the antimicrobial susceptibility, as well as the importance of test‐of‐cure after the infection, in order to see if the infection has been eradicated or to identify the resistance [16]. NG is a fastidious organism that requires strict transport and storage conditions to maintain viability, and minimizing the time between specimen collection and culture is critical, since delays as short as 6 h can significantly reduce the likelihood of a positive result [35]. Proper sampling technique is essential for diagnostic accuracy. While clinician collection has historically been the standard, studies have shown that patient self‐collection yields culture recovery rates comparable to those of clinician‐collected samples across all anatomical sites [36]. This validation of self‐sampling is a crucial advance, as it can expand testing access, improve patient comfort and acceptance, and facilitate more frequent screening.

Although NAAT offers superior sensitivity compared to culture‐based methods, culture remains essential for antimicrobial susceptibility testing. This distinction is illustrated by a study assessing the impact of transitioning from culture to NAAT for gonorrhea diagnosis in a clinical setting. Following the switch, a significant increase in detection rates among MSM was observed: rectal gonorrhea positivity increased from 3.9% (95% CI: 3.4%–4.5%) of 6409 tested to 8.0% (95% CI: 7.4%–8.7%) of 7470 tested, and pharyngeal positivity increased from 1.6% (95% CI: 1.3%–1.9%) of 6355 tested to 8.3% (95% CI: 7.7%–8.9%) of 7347 tested [37]. In a study involving 10,396 asymptomatic individuals who underwent multisite testing for NG, 710 samples tested positive (the majority originating from the pharynx); however, at a follow‐up visit a median of 1 day later, culture confirmation was successful in only a fraction of cases: 21.7% of pharyngeal infections, 37.0% of urogenital infections, and 66.9% of rectal infections [38]. Despite the diagnostic gains of the NAAT testing, culture‐based methods remain indispensable for monitoring emerging resistance patterns and guiding treatment recommendations.

Genome‐wide association studies (GWAS) have identified genetic loci associated with reduced susceptibility to specific antibiotics, paving the way for molecular diagnostics that predict resistance. Such assays are already in clinical use: testing for mutations at *gyrA* codon 91 can predict ciprofloxacin susceptibility, allowing clinicians to use ciprofloxacin as treatment [39]. Additional associations continue to emerge [40], including *penA* alleles linked to penicillin resistance, *rpsJ* codon 57 associated with tetracycline susceptibility [41], and *rplD* G70D linked to macrolide resistance [42]. While not all are yet in routine clinical use, these molecular markers offer the potential for rapid, point‐of‐care, personalized diagnostics to guide targeted therapy, particularly valuable while awaiting culture and susceptibility results.

### 4.4 Partner Notification

All sexual partners of a patient with confirmed gonorrhea within the preceding 3 months should be notified, tested, and treated if found positive [16]. Partner notification can be challenging in clinical practice, and some physicians may feel uncomfortable initiating these conversations [43]; however, in high‐income settings, digital technologies offer valuable tools to support and expand partner notification efforts, enhancing reach, identifying additional positive cases, and ultimately contributing to reduced transmission. Mathematical modeling suggests that a 10% increase in partner notification among MSM could lead to a measurable reduction in gonorrhea incidence, with an estimated incidence rate ratio of 0.98 [44].

Internet‐based STI testing services, which are increasingly utilized in modern healthcare, can be similarly effective in facilitating partner notification as traditional clinic‐based approaches: in a Canadian study, 97.2% of patients diagnosed with gonorrhea and/or CT through internet‐based services engaged in partner notification, compared to 96.5% of those managed by STI clinic nurses [45]; however, engagement varies: in England, it has been reported that only 21% of gonorrhea patients agreed to participate in an anonymous digital partner notification platform [46].

Expedited partner therapy (EPT), the provision of treatment to the sexual partners of individuals diagnosed with gonorrhea without requiring prior medical evaluation, has proved to be effective in reducing transmission in a 22‐month trial conducted in Washington State, where the distribution of cefixime 400 mg plus azithromycin 1 g to partners led to a decrease in gonorrhea incidence from 59.6 to 26.4 cases per 100,000 population [47]. However, growing concerns about antimicrobial resistance, particularly given the absence of susceptibility testing in EPT, have limited its broader adoption and endorsement.

### 4.5 Gonorrhea Trends in High‐Resource Settings

Despite the availability of advanced diagnostics, robust screening programs, and established antimicrobial resistance surveillance, both the incidence of infection and the prevalence of resistant strains continue to rise in high‐income settings. This trend underscores that technological capacity alone is insufficient to control the epidemic and suggests that a complex interplay of behavioral, biological, and healthcare‐related factors is at play. Global travel and mobility, including for sexual encounters, facilitate the rapid cross‐border dissemination of resistant strains; partner networks with inconsistent condom use contribute to high transmission rates, the widespread adoption of highly sensitive NAATs has improved case detection, and the use of DoxyPEP may be inducing resistance in gonococcal strains.

## 5 Gonorrhea in Limited‐Resource Settings

**FIGURE 2:**
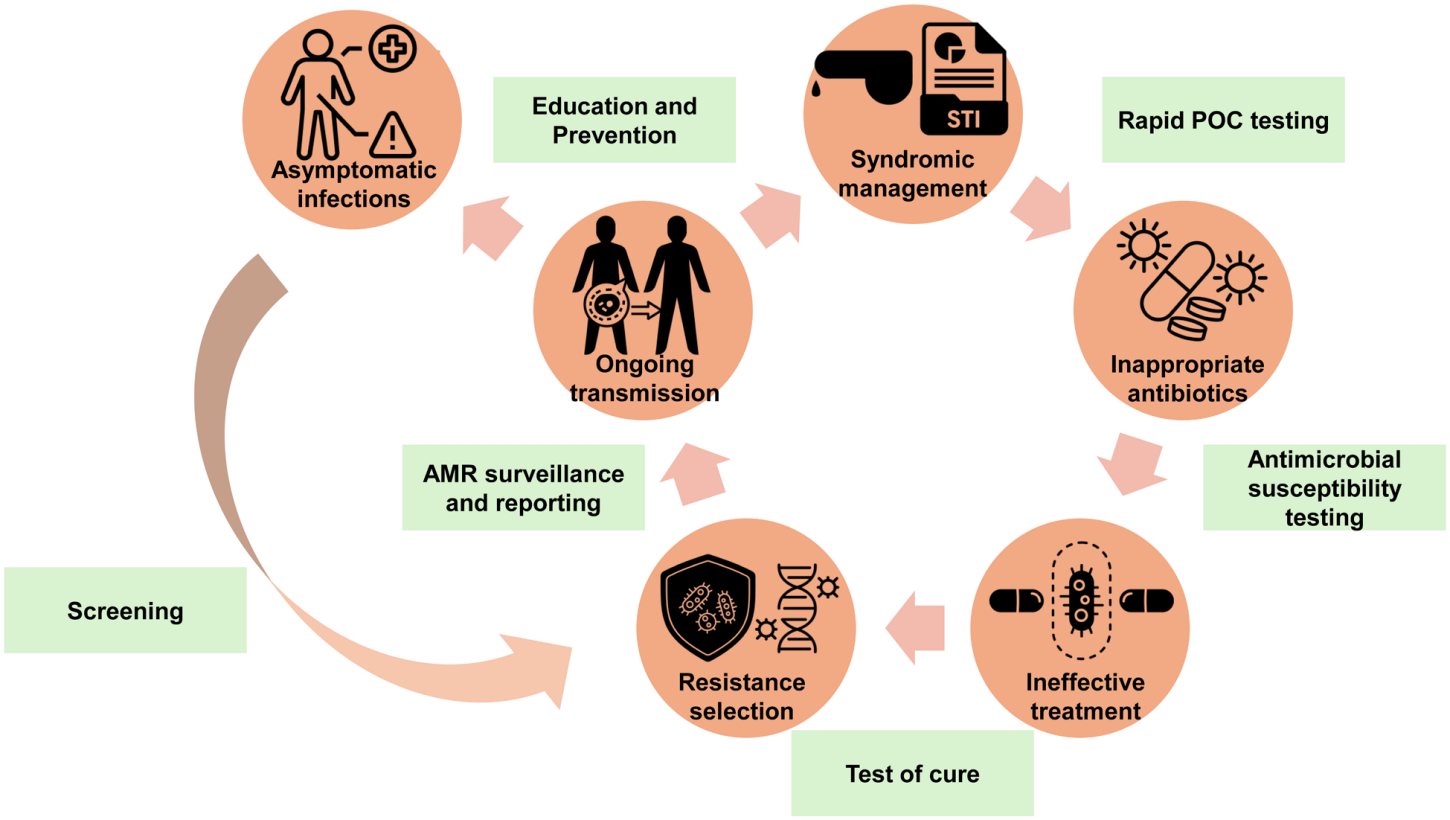
Cycle of resistance amplification in low‐resource settings. Syndromic management without diagnostics can lead to inappropriate antibiotic use, ineffective treatment, and resistance selection, fueling ongoing transmission. Key intervention points such as rapid testing, test‐of‐cure, surveillance, and prevention are shown as opportunities to break the cycle and improve gonorrhea control. AMR, antimicrobial resistance; POC, point‐of‐care; STI, sexually‐transmitted infection.

In limited‐ and no‐resource settings, such as rural clinics, low‐income health systems, and even crisis zones like refugee camps, gonorrhea management is severely hindered by the lack of diagnostic tools, AMR testing, and consistent drug supply. Syndromic management, while widely used, risks misdiagnosis and inappropriate treatment, potentially worsening antimicrobial resistance (Figure 2). These regions face multiple constraints, including limited access to culture or molecular diagnostics, restricted availability of second‐line therapies, and a high burden of sexually transmitted coinfections.

### 5.1 Syndromic Approach

Since the 1980s, the WHO has promoted syndromic case management (SCM) as a pragmatic approach in the absence of laboratory diagnostics. SCM relies on identifying symptom clusters such as urethral or vaginal discharge and treating patients empirically to cover the most likely pathogens. While effective for reducing some symptomatic STIs, SCM has notable shortcomings, especially for asymptomatic or non‐specific infections, since it does not address an important part of the infections that remain untreated; also, studies have shown that the diagnostic accuracy of SCM for CT and NG is limited [48], often leading to overtreatment and contributing to antimicrobial resistance [49]. Broad empirical use of antimicrobials such as azithromycin has been linked to the global rise in resistance among STI pathogens [50], raising concern over the long‐term effectiveness of such strategies.

Syndromic management of urethral discharge is primarily targeting CT and NG, although other pathogens such as *Mycoplasma genitalium* and *Trichomonas vaginalis* may also be involved. In contrast, syndromic management of cervical discharge in women has significant limitations, including a high rate of overtreatment (e.g., for endogenous vaginitis or bacterial vaginosis) and missed infections. Diagnostic approaches based solely on risk assessment yield low sensitivity (27.9%; 95% CI: 24.7%–31.1%) and specificity (57.0%; 95% CI: 56.1%–58.0%). While incorporating speculum examination, wet mount microscopy, and Gram staining can substantially improve sensitivity (90.1%; 95% CI: 85.8%–94.4%), specificity remains low (35.3%; 95% CI: 33.4%–37.1%) [48].

Despite its limitations, the syndromic approach remains a valuable tool for reducing the burden of STIs in resource‐limited settings, and reported sensitivities for urethral discharge management vary, but have reached levels as high as 88% in studies conducted in India, where national guidelines recommend empiric treatment with cefixime 400 mg and azithromycin 1 g for this syndrome [51].

The practice of empirical treatment, without AMR testing, is known to result in increasing reports of antimicrobial resistance, including resistance to azithromycin, ciprofloxacin, and even ceftriaxone, which is often the last resort agent. For example, in Australia, high‐level azithromycin resistance (MIC ≥ 256 mg/L) has been reported with growing frequency [52]. As mentioned previously, in the UK, the emergence of azithromycin‐resistant strains has prompted a shift from dual therapy to ceftriaxone monotherapy [50].

In settings lacking AMR monitoring infrastructure, DoxyPEP may not be advisable, or should be introduced cautiously within pilot programs.

### 5.2 Point‐of‐Care Testing

Limited access to reliable diagnostic tools remains a major bottleneck in controlling gonorrhea in low‐resource settings. Point‐of‐care testing, such as near‐patient NAAT for CT and NG, can deliver results within 90 min and has been approved by the United States Food and Drug Administration (FDA). These tests demonstrate high diagnostic accuracy using urine, vaginal, or cervical swabs, with sensitivities exceeding 95% and specificities > 99.8% across all specimens, compared to laboratory‐based NAAT reference tests [11]; they also demonstrate acceptable performance for extragenital samples (with a sensitivity of 89.7% and specificity of 99.6% for rectal *N. gonorrhoeae* detection, and 75.9% and 98.8%, respectively, for pharyngeal specimens) [53]; additionally, the samples can be self‐collected.

Although such point‐of‐care tests for NG (and CT) require electricity and calibration, they can be implemented in basic infrastructure settings: promising outcomes have been reported in remote populations, including Aboriginal communities in Australia, antenatal clinics in Papua New Guinea, and among HIV‐infected pregnant women in South Africa [48]. In settings where laboratory resources are limited, point‐of‐care testing plays an increasingly vital role in expanding diagnostic access: near‐patient testing can be conducted across diverse clinical sites, enabling timely diagnosis and treatment, and centralized laboratories can serve primarily as reference centers for quality assurance, validation, and confirmatory testing [54].

Risk stratification—prioritizing high‐risk groups for screening or more intensive monitoring—may be a mitigation strategy for limited resources distribution; however, its implementation is inconsistent due to infrastructure limitations.

Other recent innovations offer hope: a novel lateral flow immunoassay for NG, a nonmolecular rapid point‐of‐care test, showed high sensitivity (96.1% in urine; 91.7% in vaginal swabs) and specificity (> 96%), meeting WHO target product profile requirements for low‐cost, point‐of‐care use [55]. The test's affordability (under USD 3) and rapid turnaround (under 30 min) make it a promising tool in decentralized contexts.

Although whole genome sequencing remains expensive, simplified and portable sequencing platforms, such as those using nanopore technology (e.g., Oxford Nanopore's MinION) may enable its broader use, including in remote settings, for both surveillance and personalized treatment strategies [56].

### 5.3 Populations in Crisis

Severely resource‐limited settings face significant challenges in the diagnosis, treatment, and overall management of gonorrhea.

#### 5.3.1 Inmates

In a juvenile detention setting in Hawaiʻi, USA, an STI screening program was implemented for incarcerated youth aged 12 to 17 years between 2014 and 2017: testing acceptance rates differed significantly by sex, with only 27.8% of males consenting to testing compared to 67.4% of females (*p* < 0.0001). Despite substantial positivity rates, 16.4% of tested females (*n* = 372) were positive for CT and 7.3% for NG, and among tested males (*n* = 461), 6.9% had CT and 2.6% had NG; treatment completion was suboptimal: only 50% of infected females and 38.6% of infected males received treatment. This was largely attributed to reluctance to accept syndromic treatment prior to test results and to logistical barriers such as discharge from detention before follow‐up could be completed [57]. These gaps represent missed opportunities for effective STI management before reintegration into the community. In another study involving 557 youth who consented to STI testing, 12.01% of males and 14.01% of females tested positive for NG, CT, or both via urine‐based screening. In this setting, treatment uptake was significantly higher, with 74.7% of those who tested positive receiving treatment and no recorded refusals; however, 25.4% of positive individuals were released from custody before results were available, underlining the ongoing challenge of timely treatment in transient or incarcerated youth populations [58].

#### 5.3.2 Migrants

The data on migrants regarding gonorrhea infections remains scarce. A recent systematic review analyzing over 500,000 refugees and asylum seekers (RAS) has highlighted significant regional disparities in the burden of STIs, finding the highest prevalence of gonorrhea (1.53%, 95% CI: 0.16%–1.09%) among RAS originating from sub‐Saharan Africa [59]. Smaller, localized studies, for example, a 2‐year retrospective analysis (2018–2019) of 12,594 patients attending Malta's public sexual health clinic, reported a gonorrhea prevalence of 2.9% (*n* = 359), and among the 2064 patients from non‐EU/European Free Trade Association (EFTA) countries, the likelihood of an STI diagnosis was significantly higher compared to EU/EFTA citizens (odds ratio [OR] = 1.74; 95% CI: 1.53–1.98) [60]. In a cohort of 143 non‐European migrants attending the same clinic, six cases of NG urethritis were identified, with an overall STI prevalence of 73.1%. Notably, 33.8% of participants reported never using condoms, and six individuals disclosed involvement in sex work [61]. Migration presents specific health challenges and is recognized as a global public health priority, particularly in relation to sexual health and the burden of STIs.

According to evidence‐based recommendations for the care of migrants and asylum seekers in Europe, polymerase chain reaction (PCR) testing for CT and NG is advised for asymptomatic individuals with risk factors during second‐line reception [62]. Migrants are particularly vulnerable to sexual health risks and sexual violence, and their care should include comprehensive STI testing, such as gonorrhea PCR from urine, pharyngeal, and rectal sites based on sexual practices. A thorough assessment should also explore potential sexual abuse or female genital mutilation, evaluate substance use, provide vaccination catch‐up, and address mental health needs [63]. French guidelines for migrants from Ukraine recommend gonococcal screening via urine or vaginal self‐sampling for sexually active individuals under 25 years of age, and for others when risk factors are present [64]. These guidelines were developed in 2022 specifically in response to the large‐scale displacement from Ukraine, highlighting how public health guidance is adapted to acute humanitarian crises.

#### 5.3.3 Wars and Humanitarian Crises

Data on the impact of war and humanitarian crises on gonorrhea incidence is limited; however, STI rates are known to rise in such contexts, with contributing factors including forced migration, disrupted access to healthcare and prevention services, and increased risk of sexual violence [65]. During the armed conflict in Ethiopia, Tigray, which severely damaged the healthcare infrastructure, 927 of 2575 hospital patients were diagnosed with an STI, the most common syndromes being vaginal discharge (65.9%), pelvic inflammatory disease (16.2%), and urethral discharge (13.4%) [66]. Interestingly, a study of high school students in New Orleans found a significant increase in gonorrhea prevalence following Hurricane Katrina, particularly in the affected neighborhoods: prevalence increased from 2.3% (95% CI: 1.3%–4.6%) among 346 students before the hurricane to 5.1% (95% CI: 3.1%–8.2%) among 333 students afterward (*p* = 0.027) [67]. The inadequate sexual and reproductive health services during such humanitarian crises can determine an increased morbidity and mortality, particularly for females [68].

Isolated cases of ceftriaxone shortages have been reported in the literature, attributed to surges in intra‐abdominal infections [69]. In crisis settings, antibiotics may become scarce or entirely unavailable, a devastating scenario given the serious consequences of untreated gonorrhea. This shows the importance of ensuring that humanitarian responses include provisions for antibiotic supply and distribution.

Furthermore, it has become increasingly clear that antimicrobial resistance must be a priority within humanitarian initiatives; therefore, strategies for the rational use and stewardship of antibiotics should be embedded in these efforts to ensure both immediate care and long‐term effectiveness of treatments [70].

## 6 Future Horizons: Novel Treatments and Vaccination

As the threat of untreatable gonorrhea grows, the development of new control strategies is a global health priority.

Two novel oral antibiotics have demonstrated efficacy in Phase 3 trials: gepotidacin, a first‐in‐class triazaacenaphthylene antibiotic, and zoliflodacin, a spiropyrimidinetrione antibiotic, both having mechanisms of action different from the existing drugs. Both antibiotics have shown non‐inferiority to standard dual therapy in clinical trials and are currently under regulatory review [71, 72].

In parallel, an existing vaccine is repurposed for preventing gonorrhea infections. There is evidence that the serogroup B meningococcal vaccine (4CMenB) provides partial cross‐protection against NG, due to the high genetic similarity between the two *Neisseria* species [73]. In August 2025, England's National Health Service launched the first publicly funded gonorrhea vaccination program, offering the 4CMenB vaccine to individuals at high risk of infection [74].

## 7 Conclusions

In high‐resource settings, gonorrhea management benefits from advanced diagnostics, proper surveillance systems, and broad access to effective treatment, national programs for routine screening of high‐risk populations, test‐of‐cure protocols, and antimicrobial susceptibility testing. These regions often serve as early adopters of innovations such as DoxyPEP and genome‐wide association analyses and contribute robust data to surveillance networks like Euro‐GASP. However, despite these advantages, rising infection rates and emerging resistance underscore that even well‐resourced health systems face evolving challenges in controlling NG.

Effective management of gonorrhea in limited‐resource settings requires a multifaceted approach, combining revised syndromic protocols supported by rapid diagnostic tests, risk stratification strategies to target high‐burden populations, judicious consideration of DoxyPEP where AMR data support its use, expansion of affordable molecular diagnostics leveraging GWAS insight, and international collaboration for data sharing and capacity‐building in AMR surveillance. Investments in diagnostic infrastructure and tailored public health strategies are critical to overcoming the challenges of managing gonorrhea where resources are scarce. Bridging these gaps is essential to safeguard treatment efficacy and curb the global spread of drug‐resistant NG.

Equitable access to gonorrhea prevention and care must extend to marginalized populations, including migrants, incarcerated individuals, and people in humanitarian crises, who often face the highest barriers to diagnosis and treatment. Expanding routine surveillance and molecular resistance testing to these settings is essential for capturing the full global picture and tailoring effective interventions.

Populational movements and tourism, including sexual tourism, facilitate the interconnectedness and interchangeability of different settings. This allows modifications in NG related to AMR to be easily transported across borders and between settings, as NG effectively needs no “passport” to travel.

Achieving the WHO target of a 90% reduction in gonorrhea incidence by 2030 [1] will be highly challenging, particularly given the growing threat of antimicrobial resistance, uneven access to diagnostics and treatment, and structural disparities in STI care across global settings.

## Conflicts of Interest

George‐Sorin Tiplica received speaker fees from Abbott, Organon, Genesis Biopharma Romania SRL, Leo Pharma Romania, Antibiotice SA and BMS. Andrei Tanasov, Maciej Pastuszczak: none to declare.

## Data Availability

The data that support the findings of this study are available from the corresponding author upon reasonable request.
